# Harnessing the potential of deep eutectic solvents in biocatalysis: design strategies using CO_2_ to formate reduction as a case study

**DOI:** 10.3389/fchem.2024.1467810

**Published:** 2024-10-25

**Authors:** Marijan Logarušić, Karla Šubar, Maja Nikolić, Ana Jurinjak Tušek, Anja Damjanović, Mia Radović, Ivana Radojčić Redovniković, Polona Žnidaršič-Plazl, Wolfgang Kroutil, Marina Cvjetko Bubalo

**Affiliations:** ^1^ Faculty of Food Technology and Biotechnology, University of Zagreb, Zagreb, Croatia; ^2^ Faculty of Chemistry and Chemical Technology, University of Ljubljana, Ljubljana, Slovenia; ^3^ Institute of Chemistry, University of Graz, Field of Excellence BioHealth, BioTechMed Graz, Graz, Austria

**Keywords:** deep eutectic solvents, rational design, biocatalysis, mathematical modelling, QSAR, formate dehydrogenase, NADH, CO_2_ conversion

## Abstract

**Introduction:**

Deep eutectic solvents (DESs) have emerged as green solvents with versatile applications, demonstrating significant potential in biocatalysis. They often increase the solubility of poorly water-soluble substrates, serve as smart co-substrates, modulate enzyme stereoselectivity, and potentially improve enzyme activity and stability. Despite these advantages, screening for an optimal DES and determining the appropriate water content for a given biocatalytic reaction remains a complex and time-consuming process, posing a significant challenge.

**Methods:**

This paper discusses the rational design of DES tailored to a given biocatalytic system through a combination of experimental screening and computational tools, guided by performance targets defined by solvent properties and process constraints. The efficacy of this approach is demonstrated by the reduction of CO_2_ to formate catalyzed by NADH-dependent formate dehydrogenase (FDH). By systematically analyzing FDH activity and stability, NADH stability (both long-term and short-term stability after solvent saturation with CO_2_), and CO_2_ solubility in initially selected glycerol-based DESs, we were able to skillfully guide the DES screening process.

**Results and discussion:**

Considering trade-offs between experimentally determined performance metrics of DESs, 20% solution of choline chloride:glycerol in phosphate buffer (ChCl:Gly_80%B_) was identified as the most promising solvent system for a given reaction. Using ChCl:Gly as a co-solvent resulted in an almost 15-fold increase in FDH half-life compared to the reference buffer and stabilized the coenzyme after the addition of CO_2_. Moreover, the 20% addition of ChCl:Gly to the buffer improved the volumetric productivity of FDH-catalyzed CO_2_ reduction in a batch system compared to the reference buffer. The exceptional stability of the enzyme in this co-solvent system shows great potential for application in continuous operation, which can significantly improve process productivity. Additionally, based on easily measurable physicochemical solvent properties and molecular descriptors derived from COSMO-RS, QSAR models were developed, which successfully predicted enzyme activity and stability, as well as coenzyme stability in selected solvent systems with DESs.

## 1 Introduction

Medium engineering is one of the main components of biocatalysis engineering, which also includes substrate engineering, protein (enzyme) engineering, biocatalyst (formulation) engineering, biocatalytic cascade engineering, and reactor engineering (Sheldon and Pereira, 2017). Its purpose is to enhance the performance of biocatalysts, improve substrate solubility, and positively influence reaction equilibrium, so it has been a significant research focus for over the past 30 years (Castillo et al., 2016; Sheldon and Pereira, 2017; Sheldon et al., 2023). Accordingly, numerous enzymatic syntheses have been developed in both aqueous and non-aqueous media, including organic solvents, supercritical fluids, ionic liquids (ILs) and, more recently, deep eutectic solvents (DESs). The latter two, ILs and DESs, are of particular interest due to their high tunability as they can fulfil specific process requirements and meet “green solvent” criteria (Lozano et al., 2010; Villa et al., 2019; Panić et al., 2021; van Schie et al., 2021; Žnidaršič-Plazl, 2021a; Sheldon et al., 2023; Zhang et al., 2024). Eutectic systems have long been known as multi-component mixtures used in materials chemistry and engineering. However, in 2003 Abbott et al. coined the term “deep eutectic solvents” to describe mixtures that solidify at temperatures well below (*deeper*) than the crystallization points of their individual components, such that these compositions remain liquid even at room temperature (Abbott et al., 2003). In general, these solvents are formed by mixing two or more compounds that are normally solid at room temperature; when combined in certain molar ratios, they form a liquid solution due to molecular interactions (mainly hydrogen bonds) that lower the melting point of the mixture. In practice, DES can be easily prepared by combining hydrogen bond acceptors (HBAs) such as choline chloride and betaine with hydrogen bond donors (HBDs) such as polyols (e.g., glycerol, ethylene glycol, sorbitol), organic acids (e.g., citric, malic, oxalic acid), amino acids (e.g., alanine, proline), sugars (e.g., glucose, sucrose, trehalose) or amide urea with an atom economy of 100%. These combinations lead to different DESs, which almost always contain water to reduce the viscosity or to adjust certain properties of the DES (Hansen et al., 2021). The diversity of DES-forming components has led to many new and structurally different DESs, refining the initial definition by Abbott’s group. Recently, a (deep) eutectic solvent/system has been defined as a liquid system with eutectic properties that remains liquid at a given temperature, even if one component would normally be solid (Abranches and Coutinho, 2022). The wide range of their possible structural combinations, coupled with their sustainability and their distinctive physicochemical properties (non-volatility, non-flammability, easy and clean preparation, low to moderate toxicity), as well as the ability to fine-tune their solvent properties, make them ideal candidates for the development of efficient and sustainable processes or products (Cvjetko Bubalo et al., 2015).

In 2008, Gorke et al. published a pioneering work in which they demonstrated the potential of DESs as solvents or co-solvents for biocatalytic reactions (Gorke et al., 2008). It was shown that the media composed of urea, a strong HBD, with choline chloride, a HBA, does not denature hydrolases and even increases enzyme activity. Their study revealed that the components of DESs are significantly less denaturizing agents than expected, suggesting that the hydrogen bonding network in DESs lowers the chemical potential of the components. Since then, interest in the use of these solvents for various biocatalytic reactions has surged, as evidenced by the exponential growth in related publications over the past decade (according to Web of Science, more than 250 scientific papers have been published on this topic since 2009). DESs have shown considerable potential in enhancing biocatalysis through various mechanisms. These solvents can improve substrate solubility, act as smart co-substrates, and influence enzyme stereoselectivity. Furthermore, they are noted for their ability to improve enzyme activity and especially operational stability in reaction media (Zhang et al., 2024). In a recent study, a hydrophilic DES was used to tailor the properties of a copolymeric hydrogel utilized for enzyme immobilization. This addition not only exhibited superior mechanical properties of DES-infused hydrogel but also increased permeability to the specific substrate in a given biocatalytic reaction (Menegatti et al., 2024). Stabilization of enzymes, whether in homogeneous reactions or through enzyme immobilization, remains one of the major challenges in the development of long-term continuous biocatalytic processes. Overcoming this challenge is crucial as it can greatly increase the total turnover number and thus efficiency of biocatalysts, leading to the intensification of biocatalytic processes (Žnidaršič-Plazl, 2021b).

Despite the numerous advantages of DESs, the modification of the conventional aqueous medium with these solvents can significantly affect various aspects of a reaction, sometimes in undesirable ways. Therefore, finding the optimal DES for a given reaction remains a challenge. A promising method for rationally designing solvents for a specific biocatalytic system involves a complementary approach that integrates experimental screening with computational tools. If such an approach is successful, it would be possible to realize the full potential of these solvents while avoiding some problems associated with their use, such as high viscosity, product recovery issues and still quite unexplored methods of DES recovery and recycling. In this paper, we briefly review the current literature on DES-assisted biocatalysis and emphasize the need for strategic design of DESs for a given reaction. Furthermore, we propose and discuss complementary experimental and *in silico* methods to tackle the complexity of DES screening. Finally, this approach is illustrated by a case study of CO_2_ reduction catalyzed by formate dehydrogenase.

## 2 Optimizing the synergy between DES and biocatalysis: the quest for the ideal solvent

The synergy between DESs and biocatalysis is ideally suited for the efficient and sustainable production of commercially important products (Sheldon et al., 2023; Zhang et al., 2024). Biocatalysis, which is known to enable complex transformations with high regio-, chemo-, and enantioselectivity under mild and cost-effective conditions, could be greatly enhanced by DESs. These tunable solvents with their wide range of structural possibilities could be a particularly valuable aid in processes facing challenges such as enzyme stability/activity, cofactor stability, substrate solubility, product inhibition, and negative environmental impact. To date, a variety of DES-assisted biocatalytic reactions have been developed using versatile enzymes such as hydrolases (e.g., lipase, epoxide hydrolases, dehalogenases), oxidoreductases (e.g., alcohol dehydrogenases, laccases, peroxidases, monooxygenases), lyases (e.g., benzaldehyde lyase, phenolic acid decarboxylase), and transferases (e.g., amine transaminases) (Panić et al., 2021; Domingues et al., 2024; Zhang et al., 2024). In these reactions, DESs primarily act as (co-) solvents or additives. In some cases, they fulfil a dual function by acting as both solvents and (co-) substrates (Mourelle-Insua et al., 2019; Pätzold et al., 2019b). It should be noted that when DESs act as solvents, they are rarely used in their pure form. Instead, they are usually mixed with water, which is tightly incorporated into the DES cavities up to a proportion of about 50%. For example, Hammond et al. studied the effect of water on DES choline chloride:urea nanostructure, finding that it remains stable up to about 42% water (w/w) due to solvophobic sequestration of water into nanostructured domains around the cholinium cation. At 51% water (w/w), the structure disrupts, and water–water and DES–water interactions prevail, making the mixture resemble an aqueous solution of DES components (Hammond et al., 2017), which was later confirmed by Sapir and Harries (Sapir and Harries, 2020). Moreover, Nolasco et al. observed that in the same DES system containing <30% water (w/w), water molecules promote a strengthening of hydrogen bonds between choline chloride and urea (Nolasco et al., 2022). In biocatalysis, DES-water mixtures, whether in a water-in-DES or DES-in-water state, have been shown to be beneficial. These mixtures not only meet the enzyme’s hydration needs but also reduce the inherent viscosity of DESs to a more practical level (Sanchez-Fernandez et al., 2022).

One of the most prominent advantages of using DESs in biocatalysis is their versatility and tunability. By varying the component, an HBD and an HBA, and fine-tuning the water content, DESs can be tailored to the specific needs of biocatalytic processes. It used to be estimated that there are about 10^6^ possible structural variations of DESs (Panić et al., 2021) but with the continuous reporting of new DES components, multicomponent DESs, and various possible molar ratios of components (including water), the number of possible structural combinations appears to be unlimited. This vast chemical space offers enormous potential for solvent design, but also poses a major challenge: How to identify the “ideal” DES that fulfils the various criteria of a given biocatalytic system? Obviously, this task is time-consuming and sometimes leads to contradictory results in DES performance with respect to specific targets that are not easy to reconcile. For example, when Wu et al. tested different cholinium chloride- and cholinium acetate-based DESs with four HBDs (urea, glycerol, acetamide, ethylene glycol) in three molar ratios and at different water contents for the activity and stability of horseradish peroxidase, it was found that increasing the DES concentration as a co-solvent in aqueous media made the enzyme much more stable but less active than in the reference buffer (Wu et al., 2014). This pattern has been confirmed in numerous cases: DESs that stabilize enzymes (usually co-solvents with low water content) are often poor media for catalytic reactions due to the reduced enzyme activity and high solvent viscosities, resulting in slow reaction rates (Taklimi et al., 2023). Additionally, our extensive experience with these solvents has taught us that hydrophobic substrates are readily soluble in acidic DESs containing organic acids as HBDs or in hydrophobic DESs based on terpenes and fatty acids, which are generally less suitable media for enzymes than hydrophilic DESs based on polyols, sugar alcohols and sugars (with the exception of lipases, which work well in hydrophobic DES (Elgharbawy et al., 2023; Ma et al., 2024). All of this implies that it is crucial to strike a perfect balance, which essentially means finding the DES candidate that satisfies all relevant factors for the application, such as stabilization and activation of enzymes or improved substrate solubility, in a balanced manner.

Traditionally, navigating this vast design space to determine an optimal DES for specific applications has mostly relied on trial-and-error methods (e.g., measuring rection rates and calculating reaction yields in different DESs), without systematic exploration of the influence of DES on specific reaction targets (e.g., enzyme behavior and substrate solubility in these solvents), and with structure-property relationships using available computational tools. This has hindered the strategic design of these inherently tailored solvents. In addition, the understanding of intermolecular interplay between DES components, water and reactants is still in its infancy although our comprehension of these solvents has evolved over the last decade.

**FIGURE 1:**
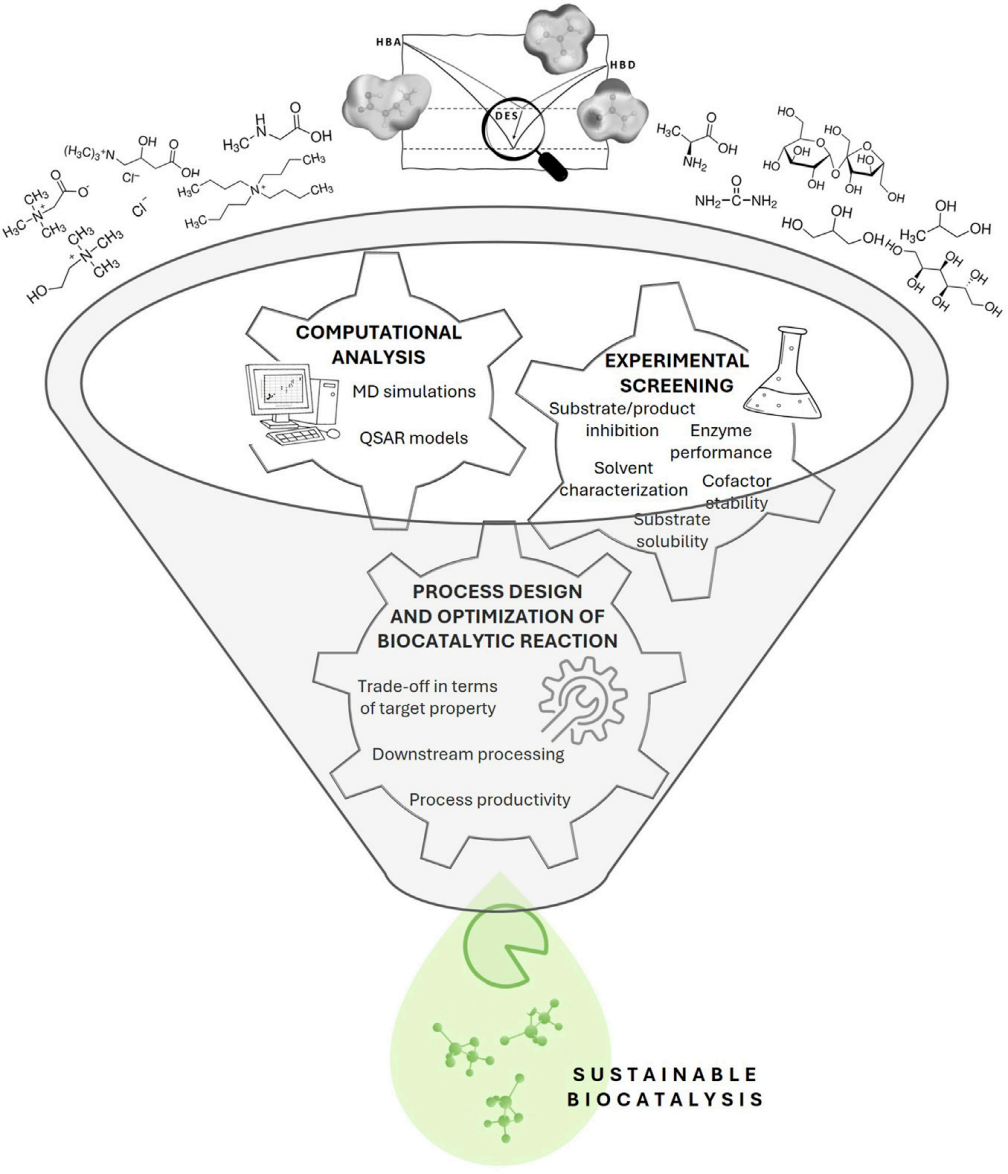
Rational design of DES for sustainable biocatalysis.

To fully exploit the potential of DESs and accelerate the design of a DES with optimal properties, several important steps are required: (*i*) Obtaining a thorough understanding of the DES structure at the molecular level and its corresponding properties; (*ii*) Understanding the interactions of DES with reacting compounds (biocatalyst, substrate(s)/product(s), cofactor(s), co-substrate(s)) at the molecular level by combining experimental and computational approaches; (*iii*) Comprehensive experimental screening of promising DES candidates with respect to factors relevant to the process of interest (solubility and stability of reacting compounds, enzyme activity, and product/substrate inhibition); (*iv*) Utilizing computational tools to establish relationships between DES composition and desired properties, and possibly develop predictive Quantitative Structure-Property Relationships (QSPRs); (*v*) Development of sustainable methods for product recovery, and recycle/reuse of DES (Figure 1). In addition to the above steps, green chemistry principles should be considered when selecting DES, including consideration of the environmental footprint of the solvent, cost and ease of recycling (Abildskov et al., 2013). By favoring sustainable and non-toxic DESs, we could minimize environmental impact while optimizing the biocatalytic processes.

Molecular dynamics simulations have proven to be powerful in understanding thermodynamic and transport processes in DESs at the atomic level, providing insights into fundamental phenomena that may not be accessible through experiments (Bittner et al., 2024). Another computational tool, the Conductor-like Screening Model for Real Solvents (COSMO-RS), provides a computational approach to generate σ-profiles (molecular descriptors) of DESs, which provide essential information on hydrogen bonding, and electrostatic and dispersion interactions in solutions (Klamt, 2005). These descriptors enable the quantification of structural changes and are widely used for high-throughput screening of DES candidates regarding the solubility of organic and inorganic molecules in DESs, but are also very useful in development of QSPR models and machine learning to predict the physicochemical properties of DESs (Benguerba et al., 2019; Lemaoui et al., 2020; Lemaoui et al., 2022). Recently, we developed a robust and reliable QSPR model based on artificial neural networks to predict the ability of DESs to stabilize dehydrogenase (Radović et al., 2024). Thus, we have shown for the first time that such models with their high prediction accuracy provide a reliable means for *in silico* screening of DESs, obviating the need for labor-intensive experimental screening and paving the way for rational design of tailored solvents. In general, mathematical and simulation models allow for the exploration of a vast chemical space of DESs that would be impossible to capture experimentally, making them essential for optimizing these solvents for industrial applications. However, accurately modeling and simulating DESs at the molecular level is challenging due to the simultaneous occurrence of multiple interactions (Lemaoui et al., 2020). Concerning the ecological footprint of DESs, they generally exhibit low to moderate toxicity to vertebrates, invertebrates, and various animal cell lines (Lomba et al., 2021). Several authors reported that DESs produced from natural metabolites can be classified as “biodegradable” according to OECD guidelines (Radošević et al., 2015; Torregrosa-Crespo et al., 2020). This means that as long as natural metabolites such as choline, betaine, polyols, sugars, and amino acids are used in the preparation of DESs, these solvents are expected to have a low environmental impact. In terms of cost, DESs are generally easy and inexpensive to prepare from readily available and renewable materials with 100% atom economy. Depending on their primary constituents, the cost of DESs ranges from €7 to €100 kg^−1^, which is comparable to organic solvents (Panić et al., 2021). The cost flexibility allows solvent selection that matches the price of the product, making DESs an attractive option for large-scale applications (Rente et al., 2022).

It should be noted that the downstream processes of the biocatalytic reactions in DESs, which are often the most complex and costly part of the whole process, are not yet sufficiently addressed. One of the major challenges in DES-assisted biocatalytic reactions is the isolation of the product as well as the regeneration and reuse of the DES. In this context, an advantageous property of DESs — their non-volatility — becomes a problem unless the products are volatile and can be removed by evaporation, as has already been shown for the recovery of butyl butyrate from ILs (Pohar et al., 2012). Due to their low vapor pressure, it is practically impossible to remove DESs by evaporation. Therefore, various techniques have been proposed for the recovery of target compounds and recycling of DESs, including liquid-liquid extraction with a different solvent, solid-liquid extraction with macroporous resins, and the use of antisolvents (Panić et al., 2021; Zhang et al., 2024).

In this field, two studies stand out as they offer new perspectives for downstream processes with these solvents. First, Maugeri et al. showed the separation of alcohol and ester in DES after kinetic resolution, with the ester forming a separate phase, a viable method when the product or substrate is insoluble in DES (Maugeri et al., 2012). Secondly, in the study by Pätzold et al., DES compounds (menthol and dodecanoic acid) acted simultaneously as substrates and reaction solvent in lipase-catalyzed esterification for the synthesis of (-)-methyl dodecanoate, where the product was separated from the DES reaction mixture by a vacuum distillation step, and a second esterification reaction could be performed with the recovered (-)-menthol (Pätzold et al., 2019a). Both studies show that through innovative thinking in downstream processing, the unique properties of these solvents can be utilized, making them very attractive for sustainable biocatalysis.

## 3 Navigating DES screening complexity: enzymatic CO_2_ conversion to formate as a case study

Given the demonstrated potential of DESs as versatile media for biocatalysis, our study aimed to illustrate effective strategies for screening these solvents using the case of formate dehydrogenase (FDH)-catalyzed reduction of CO_2_.

**FIGURE 2:**
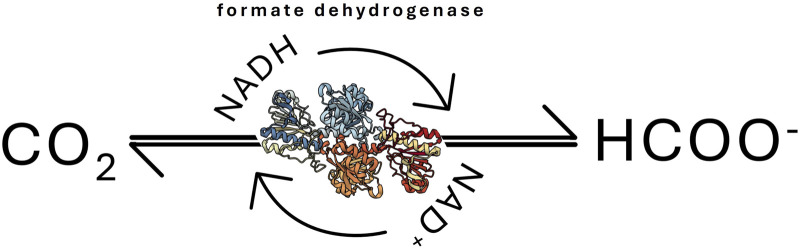
Formate dehydrogenase (FDH)-catalysed CO_2_ conversion to formate.

The reduction of CO_2_ — whether by electrochemical or (bio) catalytic means — produces formate, the first stable intermediate product in the conversion of CO_2_. The resulting formate can be further converted to valuable chemicals such as formaldehyde and methanol through additional enzymatic reactions involving aldehyde dehydrogenase and alcohol dehydrogenase. However, formate is now increasingly recognized as an energy source in its own right (Reda et al., 2008; Villa et al., 2023). It is known that the enzyme FDH is able to reduce CO_2_ to formic acid using a coenzyme such as NADH or NADPH (Figure 2) (Villa et al., 2023). The complexity of this reaction arises from several limitations: (*i*) the low concentration of CO_2_ available for the enzyme, leading to a low reaction rate, (*ii*) the relative instability of FDH and the NADH coenzyme in aqueous environments, (*iii*) the acidification of the reaction medium, firstly by the dissolution of CO_2_ and secondly by formic acid formation, can cause further FDH inhibition and NADH degradation (Zhang et al., 2018; Calzadiaz-Ramirez and Meyer, 2022).

Removing these limitations is critical to making FDH-catalyzed reduction of CO_2_ to formate a practical and economically viable process for industrial and environmental applications. Among other approaches, switching to unconventional media could help solve the problems stated above. Zhang et al. were the first to show that ILs can act as cosolvents for enhanced conversion of CO_2_ to methanol catalyzed by NADH-dependent FDH: the 67.1% conversion achieved in 1-butyl-3-methylimidazolium tetrafluoroborate was more than twice that in phosphate buffer (24.3%) (Zhang et al., 2018). Later, the same group demonstrated the advantages of introducing DES, L-serine:glycerol at a molar ratio of 1:6, into the electro-enzymatic conversion of CO_2_ and achieved a 16-fold higher reaction yield compared to the control reaction in the buffer (Zhang et al., 2022).

Instead of the traditional approach of performing the reaction and then testing for suitability, which would be extremely complex given the vast number of possible DES candidates, here we set a performance targets defined by solvent properties and process constraints. By identifying DES that fulfil these predefined goals in a balanced way, we can significantly reduce the number of trials required and allow for a more strategic allocation of resources (Abildskov et al., 2013). Based on the above considerations, the design of the solvent system with DES was divided into several phases. First, we narrowed down the list of DES candidates based on the data available in the literature on dehydrogenase performance in these solvents as well as their sustainability attributes. Then, to gain a better understanding of DES potential in overcoming the limitations associated with the reaction, the influence of selected DESs on FDH activity and stability, NADH stability, and CO_2_ solubility was systematically investigated. A computational analysis was further performed to gain insight into the DES structure and the corresponding physicochemical properties affecting the measured parameters. For the most promising DES candidates, further experiments were performed to investigate NADH stability under acidification at CO_2_ saturation. Finally, considering the trade-offs between the results in different solvent systems with DESs in terms of performance objectives, the most suitable DES candidate was proposed. This solvent system was then tested for the reaction and compared with the results in the reference buffer. At this point, it should be mentioned that for the purpose of this study, we excluded *in situ* cofactor regeneration, e.g., based on chemical, electrochemical, photochemical, or enzymatic processes (Wichmann and Vasic-Racki, 2005) to maintain methodological clarity and to avoid potential confounding effects from new reactants.

### 3.1 DESs selection and characterization

Ensuring the stability of enzymes is crucial for their industrial implementation, as it increases operational stability, prolong activity and thus increases total turnover number, and improve cost-efficiency of the process (Žnidaršič-Plazl, 2021b; Woodley, 2022). To systematically evaluate DES candidates for performance goals, we first narrowed the list to those documented in the literature as beneficial for dehydrogenases, focusing specifically on those known to stabilize FDH. Studies suggest that polyol-based DESs, containing either choline chloride or betaine as HBA, are the most effective stabilizing media for various dehydrogenases, including FDH (Bittner et al., 2022; Gajardo-Parra et al., 2023). First, seven polyol-based DESs with either choline chloride or betaine as HBA were tested at three water contents (up to 50%, w/w) for their ability to stabilize FDH. It was confirmed that glycerol-based DES containing both tested HBAs are optimal candidates for stabilizing the enzyme upon prolonged incubation at room temperature (data not shown).

Glycerol-based DESs were recently demonstrated to stabilize NAD coenzymes (Radović et al., 2022). Besides, Leron and Li (2013), Leron et al. (2013) and Biswas et al. (2023) reported that among several choline chloride-based DES containing urea, ethylene glycol, and glycerol as HBDs, the one with glycerol had the highest CO_2_ solubility. The above studies also emphasize that water plays a crucial role in enzyme performance and CO_2_ solubility. Finally, concerning the toxicological footprint, glycerol-based DES are considered non-toxic and biodegradable (Radošević et al., 2015).

Based on the above considerations, we selected two glycerol (Gly)-based DESs, with choline chloride (ChCl) or betaine (B) as HBA in a molar ratio of 1:2, and prepared the corresponding solutions in water (10%–90%, w/w). In parallel, we also prepared solutions in 50 mM potassium phosphate buffer (pH 7.5) to keep the pH close to the enzyme’s optimal value and to possibly prevent a pH drop when CO_2_ is added to the reaction medium. As previously mentioned, DESs diluted with more than 50% water (w/w) can be considered aqueous solutions of DES components (Hammond et al., 2017). Nevertheless, these mixtures were included in the study, as a high water content within DES is often essential for enzymes to sustain their catalytic activity (Taklimi et al., 2023).

A total of 20 DES-based solvents were prepared and characterized for their physicochemical properties (pH, density, and viscosity) relevant to the reaction (Table 1). As expected, the densities and viscosities of the DES aqueous solutions were strongly influenced by the water/buffer content, peaking at mixtures with 10% water (up to 1.21 g cm^*-*^³ and 353.70 mPa s for B:Gly_10*%*W_, and 1.17 g cm^*-*^³ and 82.63 mPa s for ChCl:Gly_10%B_). In general, B:Gly-based mixtures were denser and more viscous than their ChCl:Gly-based counterparts. All mixtures tested had pH values ranging from 5.3 to 9.2, with betaine-based mixtures being more acidic than ChCl-based ones. Dissolving DES in buffer generally maintained the solutions at pH values between 7.5 and 8. All DES mixtures remained stable for 3 months under laboratory conditions and showed no signs of contamination or precipitation.

**TABLE 1:** List of DES solutions in water/buffer (10%–90%, w/w) and buffer (50 mM potassium phosphate buffer pH 7.5) used for experimental screening, together with physicochemical properties and corresponding concentration of dissolved CO_2_ (*c*_s_), pH of CO_2_ saturated solutions (pH*), first-order degradation rate constant of NADH in CO_2_ saturated solutions (*k*_NADH_*), and residual FDH activity (*A*_*Res*_) (*c*_*FDH*_ = 12.8 mg mL^-1^, *t* = 14 days, T = 30°C).

		Abbrev.	pH	*η* (mPa s)	*ρ* (g cm^-3^)	*c*_s_ (mg L^-1^)	*k*_NADH_* (min^-1^)	pH*	*A_Res_*_(%)_
			Physicochemical properties			CO_2_ solubility and NADH stability			FDH
Choline chloride-based DESs	Water dissolutions	ChCl:Gly_10%w_	7.51	77.88	1.17	282	0.004	6.95	0.0
		ChCl:Gly_30%w_	6.60	18.98	1.14	480	0.000	6.03	47.6
		ChCl:Gly_50%w_	7.12	4.16	1.09	702	0.009	5.68	42.1
		ChCl:Gly_80%w_	6.48	2.27	1.03	825	0.009	4.89	52.0
		ChCl:Gly_90%w_	6.25	1.48	1.02	965	0.000	4.8	0.0
	Buffer dissolutions	ChCl:Gly_10%B_	9.19	82.63	1.17	465	0.000	6.88	0.0
		ChCl:Gly_30%B_	8.75	19.61	1.14	727	0.000	6.89	95.1
		ChCl:Gly_50%B_	8.33	5.74	1.11	913	0.003	6.79	11.8
		ChCl:Gly_80%B_	7.75	2.50	1.06	1,057	0.000	6.53	70.7
		ChCl:Gly_90%B_	7.67	1.31	1.04	1,149	0.000	6.49	40.3
Betaine-based DESs	Water dissolutions	B:Gly_10%w_	6.48	353.70	1.21	311	0.000	4.73	33.3
		B:Gly_30%w_	5.96	20.86	1.15	618	0.001	4.26	70.0
		B:Gly_50%w_	5.33	8.84	1.13	829	0.006	3.87	51.5
		B:Gly_80%w_	5.29	2.27	1.04	1,101	0.008	3.75	11.9
		B:Gly_90%w_	5.47	1.31	1.03	1,112	0.005	3.77	0.0
	Buffer dissolutions	B:Gly_10%B_	7.85	219.05	1.21	509	0.003	6.39	60.0
		B:Gly_30%B_	7.46	32.87	1.17	586	0.000	6.26	59.8
		B:Gly_50%B_	7.45	6.45	1.13	765	0.001	6.24	65.0
		B:Gly_80%B_	7.49	2.27	1.06	1,027	0.009	6.24	58.5
		B:Gly_90%B_	7.47	1.19	1.04	1,010	0.000	6.31	48.3
		buffer	7.50	1.39	1.02	1,029	0.003	6.49	0.0

Abbreviations: choline chloride (ChCl), betaine (B), glycerol (Gly).

**FIGURE 3:**
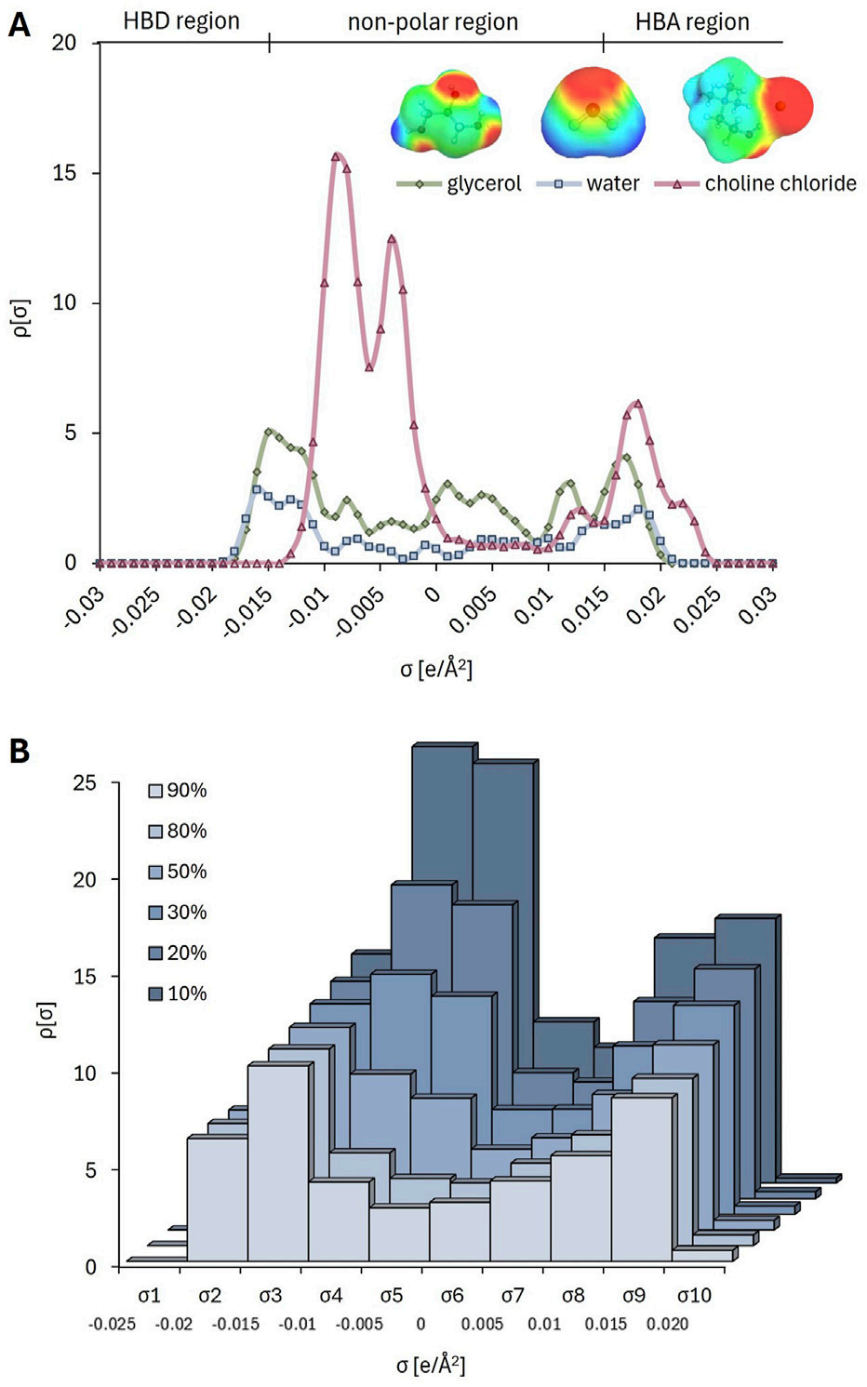
σ-surface **(A)** and *σ*-profiles **(B)** of individual DES constituents (choline chloride, glycerol and water), and σ-profile of corresponding DES solutions in buffer (10%–90%, w/w).

For statistical analysis and mathematical modelling (Section 3.4.), the identification of a molecular representation that converts the component structures into descriptive features for numerical evaluation is essential (Venkatraman et al., 2018). An advanced and accurate molecular representation is the *σ*-profile (sigma profile), an unnormalized histogram of the screened surface charge of a molecule (Klamt, 2005). *σ*-profiles are distinguished from other representations by the fact that they capture nuanced effects such as polarizability and electron density asymmetry (Abranches et al., 2022). The *σ*-profile can be divided into three key regions: (*i*) the HBD region with negative charge densities, (*ii*) the non-polar region with nearly neutral charge densities, and (*iii*) the HBA region with positive charge densities (Figure 3A). This division is based on the fact that each atom in an HBA or HBD molecule is identifiable by a distinct peak with a specific screening charge density (*σ*) value (Lemaoui et al., 2020).

Here, the *σ*-profile of each DES-based mixture was calculated using BOVIA COSMOtherm software: the σ-profile curves for each HBA and HBD were divided into 10 regions, and the area under each region was calculated considering the molar ratios of the components and the water content (Supplementary Table S1). For glycerol, the *σ*-profile reveals peaks at negative polar coordinates (left side) corresponding to the positively polar H atoms in the -OH group, while peaks at positive polar coordinates (right side) correspond to the O atoms in the -OH group (Figure 3A) (Cheng et al., 2018). These polar regions interact with opposite polar segments in a solution. The *σ*-profile’s extension into strongly polar regions (−0.022 e/Å^2^ < *σ* < −0.01 e/Å^2^ on the left and 0.01 e/Å^2^ < *σ* < 0.013 e/Å^2^ on the right) is asymmetric, indicating that glycerol has a larger positive polarity surface area. This electrostatic misfit suggests that glycerol tends to act as a HBD and thus shows greater affinity for HBA in a solution (Cui et al., 2021). In contrast, the *σ*-profile of water extends more symmetrically into strongly polar regions, indicating its balanced ability to act as both a HBD and HBA. Finally, for ChCl, as well as for betaine (Radović et al., 2024), the strongest peak appears in the non-polar region, corresponding to the cholinium cation, followed by a peak in the HBA region, associated with the Cl⁻ anion. Figure 3A illustrates the sigma surfaces of the DES components (choline chloride, glycerol and water) generated by TmoleX19. The colors represent a calculated charge gradient, ranging from charge-deficient to charge-dense regions: HBD regions are labelled as deep blue and HBA regions as deep red on the surface. Non-polar regions are marked in green (Quaid and Reza, 2023). Figure 3B shows that even small changes in the ChCl:Gly mixture, such as increasing the water content from 10% to 90%, result in solvents with different polarity distributions. This demonstrates that the software is capable of capturing nuanced phenomena, which is crucial for exploring the chemical landscape of these solvents and understanding their potential impact on enzyme and coenzyme behavior, as well as the solubility of reaction participants. We have recently demonstrated the same ability of the software for the system betaine:ethylene glycol with 3 water proportions (10, 30, 50% water, w/w) (Radović et al., 2024).

### 3.2 Activity and long-term stability of FDH in DESs

**FIGURE 4:**
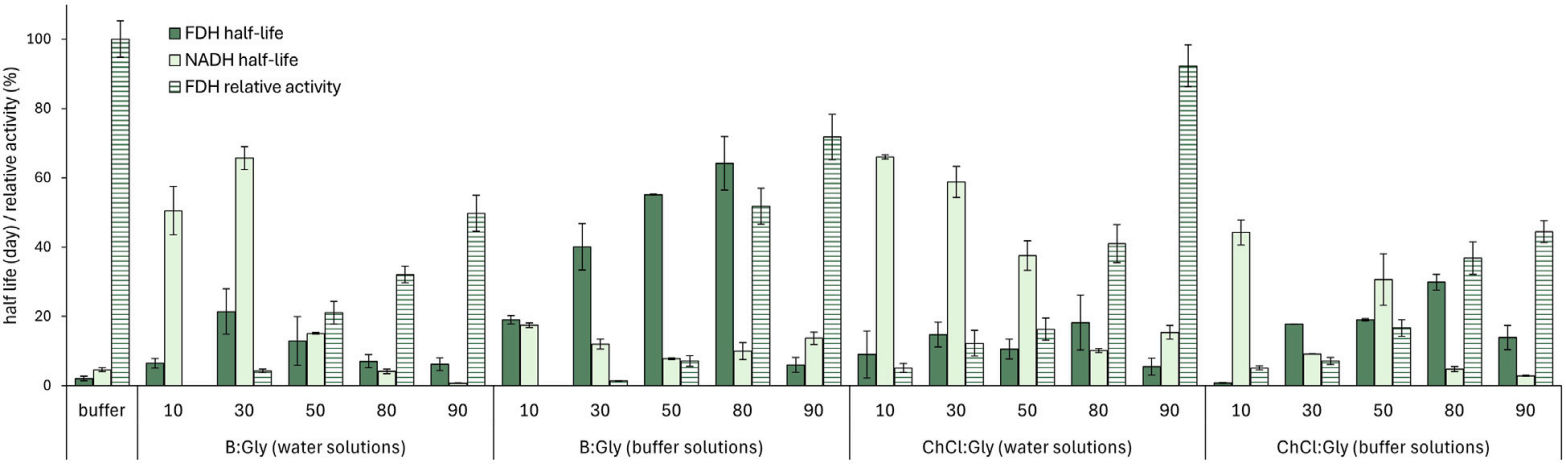
FDH relative activity (*c*_FDH_ = 1.5 mg mL^-1^; *c*_NADH_ = 0.1 mg mL^-1^; *c*_formate_ = 10 mg mL^-1^, T = 25°C) and half-life (*c*_FDH_ = 12.8 mg mL^-1^, T = 30°C), along with NADH half-life (*c*_NADH_ = 0.03 mg mL^-1^, T = 25°C) in B:Gly and ChCl:Gly solutions in water/buffer (10%–90%, w/w) and the reference 50 mM potassium phosphate buffer (pH 7.5). FDH relative activities in DES mixtures are expressed as a percentage of the activity measured in the reference buffer.

The activity of FDH in the reference buffer and in aqueous solutions of DESs at different concentrations were measured by monitoring the oxidation of sodium formate. For the stability test, enzyme solutions were incubated in selected solvent systems at 30°C for 14 days and residual enzyme activity (*A*_*Res*_) was measured at regular intervals (Table 1). Both in the reference buffer and in the mixtures with DESs, FDH inactivation followed first-order kinetics, allowing us to use this kinetic model to calculate the FDH half-life (*t*_*1/2, FDH*_) (Figure 4).

As anticipated, FDH showed little or no activity in the DES mixtures containing ≤50% (w/w) water or buffer, while the activity increased with the addition of water and peaked in the highly diluted mixtures (90% water, w/w), although the values were still lower than those observed in the buffer (residual activities, *A*_*R*_, between 44% and 92%). Interestingly, it appears that dilutions with water generally resulted in better enzyme activity than their counterparts diluted with buffer, although buffered systems are closer to the enzyme’s optimal pH of 7.5 (determined experimentally, data not shown). For example, ChCl:Gly with 90% water content (ChCl:Gly_90%W_) with a pH of only 6.3 had an *A*_*R*_ of about 92%, while its counterpart with the buffer (ChCl:Gly_90%B_) with a pH of 7.5, yielded the *A*_*R*_ of only 44.5%.

DESs with a water/buffer content in the range of 30% - 80% (w/w) showed the best ability to stabilize the enzyme, with half-lives of up to 64 days, which is much higher than in the reference buffer, where *t*_1/2,__*FDH*_ was 2.1 days (Figure 4). On the other hand, highly diluted DESs (90% water/buffer, w/w) demonstrated a stabilizing effect on the enzyme comparable to that of the reference buffer.

The stability of FDH in buffered DES solutions (80% buffer, w/w) was also significantly increased compared to the reference buffer, with *t*_*1/2*,__*FDH*_ of 65.6 days and 29.9 days for B:Gly_80%B_ and ChCl:Gly_80%B_, respectively. Again, DESs solutions with a water/buffer content of only 10% led to a faster destabilization of the enzyme. These results are consistent with previous findings that most dehydrogenases require more than 10% water content in DES to maintain their structural integrity. DESs absorb water in their hydrogen bonding network, reducing the availability of free water molecules required for enzyme hydration. This reduction in water activity can lead to dehydration and irreversible denaturation of the enzyme (Mourelle-Insua et al., 2019; Bittner et al., 2022; Radović et al., 2024). Overall, solvent systems with 80% buffer (w/w) exhibited an optimal balance between FDH activity and stability, which was particularly evident for B:Gly_80%B_, where the *A_R_* was 51.7% (Figure 4).

### 3.3 CO_2_ solubility and NADH stability in DESs

The saturated dissolved CO₂ concentrations (*c*_*s*_) in various DES solutions with water/buffer were evaluated after introduction of CO₂ at a flow rate of 100 mL min^−1^ until saturation. The results are presented in Table 1. All DESs mixtures with ≤50% water or buffer were poor media for dissolving CO_2_, with *c*_*s*_ values between 282 and 913 mg L^−1^, which is lower than those observed in the reference buffer (1,029 mg L^−1^). Highly diluted DESs led to similar CO_2_ solubilities as in the buffer, with the highest improvements observed in ChCl:Gly_90%B_ and B:Gly_90%W_, with values of 1,149 and 1,112 mg L^−1^, respectively.

To investigate the long-term stability of the NADH coenzyme, the changes in the UV-Vis absorption spectra of NADH were observed during a 14-day incubation at 25°C in the solvent systems described above. During incubation, the absorbance loss at 340 nm followed the first-order kinetics used to calculate the NADH half-lives shown in Figure 4. The results clearly show that DES composition plays an important role in the coenzyme degradation rate. Virtually all DES aqueous solutions, except ChCl:Gly_90%B_ (*t*_*1/2*,__*NADH*_ = 2.9 days) and B:Gly_90%W_ (*t*_*1/2,*__*NADH*_ = 0.8 days), stabilized the coenzyme compared to the reference buffer (*t*_*1/2, NADH*_ = 4.6 days). In general, ChCl-based DESs were more suitable for coenzyme stabilization than betaine-based DESs. This was particularly pronounced for DESs with 10% buffer content (e.g., for ChCl:Gly_10%B_, *t*_*1/2,*__*NADH*_ was 44.2 days, while for B:Gly_10%B_, *t*_*1/2, NADH*_ was 17.5 days). As evident from Figure 4, a higher DES content had a positive effect on the ability of solvent systems to stabilize the coenzyme for all DESs tested. This was most evidenced for B:Gly solutions in water, where the *t*_*1/2, NADH*_ for B:Gly_30%W_ and B:Gly_90%W_ was 65.7 and 0.8 days, respectively.

To further navigate the DES screening, the short-term NADH stability in DES aqueous solutions saturated with CO_2_ was investigated. The presence of dissolved CO_2_ not only affects the intrinsic properties of the DESs but also the behavior of the system after substrate addition. It has been previously reported that the acidification of the reaction medium by dissolving CO_2_ leads to enhanced NADH degradation, which directly affects the conversion of CO_2_ to formic acid (Zhang et al., 2018). Therefore, NADH solutions in the solvent systems described above were monitored over a period of 90 min, and the corresponding degradation constants (*k*_NADH*_) were calculated using a first-order kinetic model (Table 1). In general, buffer-diluted DESs were equally or more successful in stabilization of the coenzyme over the tested period (*k*_NADH*_ ≤ 0.003 min⁻^1^) than the reference buffer (*k*_NADH*_ = 0.003 min⁻^1^), while water-based solvent systems with DESs were poor media in this regard, especially at high water contents (*k*_NADH*_ up to 0.009 min⁻^1^). This effect is directly related to the inability of water-diluted DESs to maintain pH close to neutral. For example, after the introduction of CO_2_ into B:Gly_80%W_, pH decreased to 3.75, resulting in the highest observed *k*_NADH*_ value of 0.008 min⁻^1^. Moreover, buffered solutions of ChCl-based DESs maintained a higher pH than betaine-based DES solutions after CO_2_ saturation, resulting in complete stabilization of NADH over the time tested, except for ChCl:Gly_50%B_ (*k*_NADH*_ = 0.003 min⁻^1^).

### 3.4 Statistical analysis of data and development of mathematical models

The correlations between the physicochemical properties (pH, viscosity and density) of the DESs used, the DES descriptors (*σ*-profiles), FDH performance, long-term NADH stability and CO_2_ solubility were analyzed using the Spearman correlation matrix, which was selected due to the non-normal data distribution. The analysis confirmed our above assumptions and findings from the experimental screening: the targeted properties of the reaction affected by the DES composition led to contradictory results (Table 2). First, both FDH activity and stability in tested solvent systems showed negative correlations with NADH stability. In addition, FDH activity values demonstrated negative correlations with all analyzed variables except CO_2_ solubility, favouring aqueous/buffered media over DES solutions with lower amounts of aqueous phase. Besides, negative correlations were found also for FDH and NADH stability with CO_2_ solubility (Table 2).

**TABLE 2:** Spearman correlation matrix.

	*A*_R_	*t*_*1/2, FDH*_	*t*_*1/2, NADH*_	*c*_*S*_	*ρ*	*η*	pH	$S_{\text{mix}}^{1}$	$S_{\text{mix}}^{2}$	$S_{\text{mix}}^{3}$	$S_{\text{mix}}^{4}$	$S_{\text{mix}}^{5}$	$S_{\text{mix}}^{6}$	$S_{\text{mix}}^{7}$	$S_{\text{mix}}^{8}$	$S_{\text{mix}}^{9}$	$S_{\text{mix}}^{10}$
***A***_**R**_	1.000																
***t***_***1/2*, *FDH***_	-0.146	1.000															
***t***_***1/2, NADH***_	**-0.577**	-0.045	1.000														
***c***_**S**_	**0.887**	**-0.028**	**-0.603**	1.000													
***ρ***	**-0.939**	**0.199**	**0.635**	**-0.866**	1.000												
***η***	**-0.944**	**0.142**	**0.663**	**-0.902**	**0.942**	1.000											
**pH**	**-0.323**	**0.063**	-0.104	-0.159	**0.461**	**0.301**	1.000										
$S_{\text{mix}}^{1}$	**-0.953**	**0.146**	**0.679**	**-0.908**	**0.964**	**0.974**	**0.316**	1.000									
$S_{\text{mix}}^{2}$	**0.953**	**-0.146**	**-0.679**	**0.908**	**-0.964**	**-0.974**	**-0.316**	-1.000	1.000								
$S_{\text{mix}}^{3}$	**-0.906**	**0.101**	**0.727**	**-0.882**	**0.909**	**0.957**	0.170	**0.948**	**-0.948**	1.000							
$S_{\text{mix}}^{4}$	**-0.906**	**0.101**	**0.727**	**-0.882**	**0.909**	**0.957**	0.170	**0.948**	**-0.948**	1.000	1.000						
$S_{\text{mix}}^{5}$	**-0.953**	**0.146**	**0.679**	**-0.908**	**0.964**	**0.974**	**0.316**	1.000	-1.000	**0.948**	**0.948**	1.000					
$S_{\text{mix}}^{6}$	**-0.932**	**0.110**	**0.694**	**-0.900**	**0.948**	**0.964**	**0.286**	**0.990**	**-0.990**	**0.958**	**0.958**	**0.990**	1.000				
$S_{\text{mix}}^{7}$	**-0.906**	**0.101**	**0.727**	**-0.882**	**0.909**	**0.957**	0.170	**0.948**	**-0.948**	1.000	1.000	**0.948**	**0.958**	1.000			
$S_{\text{mix}}^{8}$	**-0.906**	**0.101**	**0.727**	**-0.882**	**0.909**	**0.957**	0.170	**0.948**	**-0.948**	1.000	1.000	**0.948**	**0.958**	1.000	1.000		
$S_{\text{mix}}^{9}$	**-0.906**	**0.101**	**0.727**	**-0.882**	**0.909**	**0.957**	0.170	**0.948**	**-0.948**	1.000	1.000	**0.948**	**0.958**	1.000	1.000	1.000	
$S_{\text{mix}}^{10}$	-0.202	0.249	-0.139	-0.072	0.177	0.099	**0.439**	0.144	-0.144	-0.144	-0.144	0.144	0.044	-0.144	-0.144	-0.144	1.000

Abbreviations: FDH relative activity (*A*_R_), FDH half-life (*t*_*1/2, FDH*_), NADH half-life (*t*_*1/2*_ *,*_*NADH*_), CO_2_ saturated dissolved concentration (*c*_*s*_), DES descriptors ( $S_{\text{mix}}^{1}$ – $S_{\text{mix}}^{10}$ ).

Correlations significant at p<0.05 are marked in bold.

The Spearman correlation matrix shown in Table 2 highlights the complex interplay between the physicochemical properties of DES (pH, density, viscosity) and the DES descriptors with the performance characteristics of FDH and the stability of its cofactor NADH. As expected, significant negative correlations were observed between FDH activity and DES density/viscosity, likely due to the lower mobility and diffusion of substrates and enzymes. Conversely, NADH stability showed significant positive correlation with the density/viscosity of the solvent systems with DESs, which can be attributed to the slower overall dynamics of the solvents providing a less detrimental environment for dissolved NADH.

FDH activity and NADH stability are significantly impacted by pH value, while for FDH stability there is no correlation with this chemical property. It is well-established that enzyme activity (Bisswanger, 2017) and NADH stability (Zachos et al., 2019) are pH-dependent. However, the consistent reports on pH-independent ability of DESs to stabilize various enzymes remains puzzling. This intriguing observation hints at other mechanisms, such as direct interactions between DES components and proteins or with nearby water molecules, potentially altering the medium’s water activity (Damjanović et al., 2024).

Furthermore, the analysis revealed that nearly all DES descriptors significantly impacted the targeted properties. Specifically, *S* ^*1*^_*mix*_ and *S* ^*2*^_*mix*_ (HBD region, medium polarity), *S* ^*3*^_*mix*_ - *S* ^*5*^_*mix*_ (nonpolar region, positive charges), *S* ^*6*^_*mix*_ – *S* ^*8*^_*mix*_ (nonpolar region, negative charges) and *S* ^*9*^_*mix*_ (HBA region) (Lemaoui et al., 2020), all demonstrated a significant influence on the properties being studied. FDH activity exhibited a negative correlation with all the descriptors, except for *S* ^*2*^_*mix*_, which showed a positive correlation. Conversely, both FDH stability and NADH stability demonstrated positive correlations with all the descriptors, except for *S* ^*2*^_*mix*_, which displayed a negative correlation. Interestingly, the analysis suggests an inverse relationship between enzyme activity and stability in DESs: solvents rich in HBA and non-polar domains stabilize the enzyme (and coenzyme), while HBD-rich solvents enhance enzyme activity but may lead to destabilization. These findings emphasize the delicate balance between enzyme activity and stability in DESs, driven by their specific compositional properties (as described by σ-descriptors). Similar interplay between the enzyme’s active and stable (but inactive) states, influenced by the water content in DESs, has been confirmed in our recent study on lysozyme behaviour in DESs based on various naturally occurring osmolytes (Damjanović et al., 2024). Insights into this relationship could guide the rational design of DESs for optimized biocatalysis applications: by performing similar statistical analyses on a larger set of DESs, it may be possible to predict an ideal *σ*-profile shape, and thereby identify or design the most suitable DES for a specific purpose.

**FIGURE 5:**
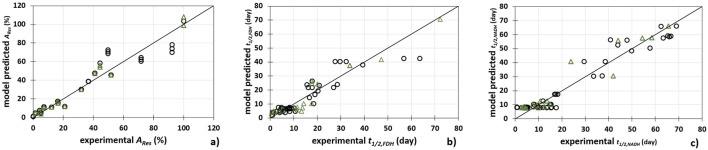
Comparison between experimental data and piecewise linear model predicted data for **(A)** FDH activity (*A*_*Res*_), **(B)** FDH half-life (*t*_*1/2, FDH*_) and **(C)** NADH half-life (*t*_*1/2,*__*NADH*_). (○) calibration data set (Δ) prediction data set.

According to discussed above, our next step was to see if it was possible to develop a simple QSAR model to summarize the relationship between the targeted properties (FDH activity, FDH stability, and NADH stability) and the DES descriptors (Figure 3; Supplementary Table S1) along with the physicochemical properties (Table 1) of the DESs using piecewise linear regression (PLR) (Figure 5). The latter is a powerful tool for modelling complex relationships in a simple and interpretable way, especially when the relationship between variables changes at a certain point. The input variables of the PLR models were selected based on the significant correlations in the Spearman correlation matrix. The relationship between the observed data and model predictions was also estimated using the coefficient of determination for prediction (*R*_*pred*_^*2*^), the adjusted coefficient of determination for calibration (*R*_*pred*_^*2*^_adj_ *)*, the root mean square error of prediction (*RMSEP*), the ratio of prediction to deviation (*RPD*) and the ratio of the error range (*RER*).

As shown in Table 3 and Figure 5, the developed models describe the experimental data with high precision. The best agreement between the experimental data and the data predicted by the model was obtained for the FDH activity (Figure 5A) (*R*_*cal*_^2^ = 0.914, *R*_*cal*_^2^_adj_ = 0.913, *RMSEC* = 7.203%, *R*_*pred*_^2^ = 0.905, *R*_*pred*_^2^_adj_ = 903, *RMSEP* = 7.379%, *RPD* = 5.589, *RER* = 18.324). On the other hand, the largest scatter between the model and experimental data was found for the FDH half-life (Figure 5B) (*R*_*cal*_^2^ = 0.829, *R*_*cal*_^2^_adj_ = 0.827, *RMSEC* = 4.775 days, *R*_*pred*_^2^ = 0.738, *R*_*pred*_^2^_adj_ = 0.731, *RMSEP* = 4.944%, *RPD* 3.292, *RER* = 10.456).

**TABLE 3:** Pricewise linear regression models for prediction of FDH stability, FDH activity and NADH stability based on the specific input variables. (Coefficient of determination for calibration (*R*_*cal*_^*2*^), the adjusted coefficient of determination for calibration (*R*_*cal*_^*2*^_adj_), the root mean square error for calibration (*RMSEC*), the coefficient of determination for prediction (*R*_*pred*_^*2*^), the adjusted coefficient of determination for calibration (*R*_*pred*_^*2*^_adj_), the root mean square error of prediction (*RMSEP*), the ratio of prediction to deviation (*RPD*),the ratio of the error range (*RER*)).

Output variable	Input variables	Model equation	*R*_*cal*_^*2*^ *R*_*cal*_^*2*^_adj_	*RMSEC*	*R*_*pred*_^*2*^ *R*_*pred*_^*2*^_adj_	*RMSEP*	*RPD*	*RER*
FDH activity	Viscosity (*X*_1_) Density (*X*_2_) pH (*X*_3_) DES descriptors 1–9 (*X*_4_-*X*_12_)	*Y*_1_ = (31.635 + 0.024 *X*_1_-81.589 *X*_2_-0.257 *X*_3_+0.382 *X*_4_+41.465 *X*_5_-98.311 *X*_6_-93.683 *X*_7_+8.822 *X*_8_+201.025 *X*_9_+123.045 *X*_10_ + 80.146 *X*_11_ + 46.359 *X*_12_) (for *Y*_1_ ≤ 30.081) + (25.079 + 35.851 *X*_1_+1782.264 *X*_2_-25.075 *X*_3_+0.080 *X*_4_+42.087 *X*_5_-99.096 *X*_6_-67.368 *X*_7_-36.899 *X*_8_+22.587 *X*_9_+29.178 *X*_10_-29.264 *X*_11_-70.624 *X*_12_) (for *Y*_1_ > 30.081)	0.914 0.913	7.203	0.905 0.903	7.379	5.589	18.324
FDH half-life	Viscosity (*X*_1_) Density (*X*_2_) pH (*X*_3_) DES descriptors 1–9 (*X*_4_-*X*_12_)	*Y*_2_ = (3.489-0.015 *X*_1_-7.078 *X*_2_-1.359 *X*_3_+0.113 *X*_4_+7.413 *X*_5_-6.365 *X*_6_-6.827 *X*_7_+2.733 *X*_8_+12.839 *X*_9_+8.943 *X*_10_ + 2.277 *X*_11_-3.795 *X*_12_) (for *Y*_2_ ≤ 14.041) + (4.429-0.229 *X*_1_-285.320 *X*_2_+11.223 *X*_3_+0.109 *X*_4_+19.063 *X*_5_+10.294 *X*_6_-10.294 *X*_7_-10.649 *X*_8_+9.620 *X*_9_+12.795 *X*_10_ + 8.317 *X*_11_ + 1.174 *X*_12_) (for *Y*_2_ > 14.041)	0.829 0.827	4.775	0.738 0.731	4.944	3.292	10.456
NADH half-life	Viscosity (*X*_1_) Density (*X*_2_) DES descriptors 1-9 (*X*_3_-*X*_11_)	*Y*_3_= (11.199 + 0.001 *X*_1_+4.581 *X*_2_+0.121 *X*_3_+21.634 *X*_4_-26.329 *X*_5_-10.074 *X*_6_-0.219 *X*_7_+29.394 *X*_8_+25.508 *X*_9_+11.581 *X*_10_-11.355 *X*_11_) (for *Y*_3_ ≤ 23.872) + (−0.217-0.175 *X*_1_+691.395 *X*_2_+0.157 *X*_3_-30.392 *X*_4_-58.475 *X*_5_-3.406 *X*_6_-7.604 *X*_7_+28.916 *X*_8_+14.168 *X*_9_+28.553 *X*_10_-24.523 *X*_11_) (for *Y*_3_ > 22.872)	0.930 0.929	4.789	0.856 0.853	6.150	3.595	14.193

According to Hussain et al., an *R* ^2^ value of 0.75 is considered significant, an *R* ^2^ value of 0.50 is considered moderate, and an *R* ^2^ value of 0.26 is considered weak (Hussain et al., 2018). Furthermore, models with *RPD* < 1.4 are considered non-reliable, those with *RPD* in the range from 1.4 to 2 are considered fair, while models with *RPD* > 2 are described as excellent models (Chang et al., 2001). Models with *RER* > 4 are acceptable for data screening, models with *RER* > 10 can be used for quality control, while models with *RER* > 15 can be used for quantification (Sim et al., 2023). Therefore, the PLR models developed for the prediction of FDH activity and NADH half-life based on *R*_pred_^2^ can be considered substantial, while the model developed for the prediction of FDH half-life can be considered moderate. Based on the *RPD* values, all three models developed can be considered reliable. And based on the *RER* values, the model developed for the prediction of FDH activity can be used for quantification (*RER* = 18.324), while the other two modes can be used for quality control. Therefore, it can be concluded that the feasibility of mathematical models for predicting targeted properties or applications of DES using easily measurable physicochemical properties and chemical descriptors, as demonstrated here, could be valuable for both industrial applications and research efforts focusing on these solvents. Additionally, utilizing these QSPR models may not only assist in predicting the properties of interest but also provide valuable insights into the relationship between the structure of the DES and its measurable properties. By analyzing how various structural features influence the targeted properties, these models can help unravel the underlying mechanisms driving behavior of biomolecules in DESs. This understanding can inform the design and optimization of DESs, leading to more effective and tailored applications in various fields.

### 3.5 Trade-off between the performance of DESs with respect to target properties

**FIGURE 6:**
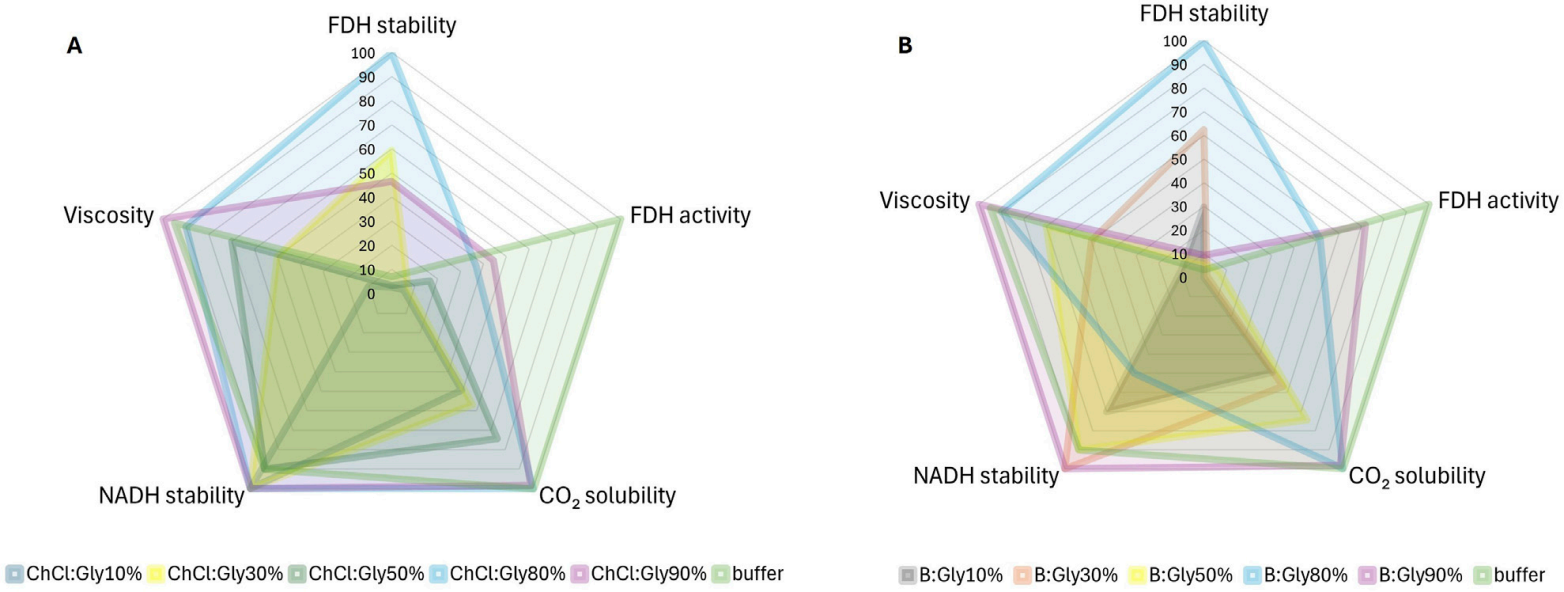
Radar plot evaluating ChCl:Gly **(A)** and B:Gly **(B)** and their corresponding solutions in buffer (10%–90%, w/w) in terms of target properties (FDH activity and stability, CO_2_ solubility in DES systems, stability of NADH after solvent saturation with CO_2_, and DES systems’ viscosity). The radar chart is bounded by the specific lower and upper limits for each target property. Ratings, ranging from 0 to 100, reflect the performance of DES-based solvents relative to the best candidate for each target property.

We have demonstrated that the evaluation of different targets related to the tested reaction often leads to contradictory results regarding the optimal DES. For example, the enzyme dissolved in B:Gly_30%B_ showed remarkable stability with a half-life of 40.1 days, while its relative activity was less than 2% of that in the buffer. This DES also showed average performance in the long-term stability of the coenzyme, with a half-life of 12.1 days. In contrast, the enzyme dissolved in B:Gly_90%B_ maintained a high relative activity, which was 71.2% of that in the reference buffer, but was one of the worst candidates for enzyme stabilization with a half-life of only 6.0 days. In general, “concentrated” DESs (<50% water/buffer, w/w), with *η* > 18 mPa s showed high efficacy in stabilizing both the enzyme and the coenzyme, but also high viscosity, which poses significant challenges for scaling up processes with these solvents. Based on these observations, it is crucial to reconcile these results by finding a DES that optimally fulfils the desired properties and thus contributes to the overall efficiency of the process. A graphical representation shown in Figures 6A, B illustrates the trade-off between the performance of ChCl:Gly and B:Gly, respectively, with different buffer proportions with respect to the targeted property values (FDH activity/stability, CO_2_ solubility, NADH stability in saturated CO_2_ solutions, and compared to the reference buffer). The radar chart is bounded by the respective lower and upper limits of each target property, with ratings, ranging from 0 to 100, reflecting the performance of DES-based solvents relative to the best candidate for each target property. At this point, DESs diluted with water were omitted due to their poor ability to stabilise NADH after acidification of the medium due to CO_2_ introduction (Table 1).

As can be seen from Figure 6A, ChCl:Gly_80%B_ has a balanced distribution across all target property values, which is crucial for the simultaneous optimization of the reaction where all design objectives are equally important. In particular, this DES, which in this case could be considered an additive rather than a solvent (Hammond et al., 2017), had the highest values for all target properties except for FDH activity, where the reference buffer resulted in the highest values. It should be emphasized that ChCl:Gly_80%B_ had an almost 15-fold higher *t*_*1/2*,__*FDH*_ value compared to the buffer and stabilized the coenzyme more effectively when CO_2_ was dissolved in the solvent system. B:Gly_80%B_ also showed similar balanced behavior to ChCl:Gly_80%B_, but was ineffective in stabilizing NADH after dissolving CO_2_.

### 3.6 FDH-catalyzed CO_2_ conversion to formate in the most promising solvent system

**FIGURE 7:**
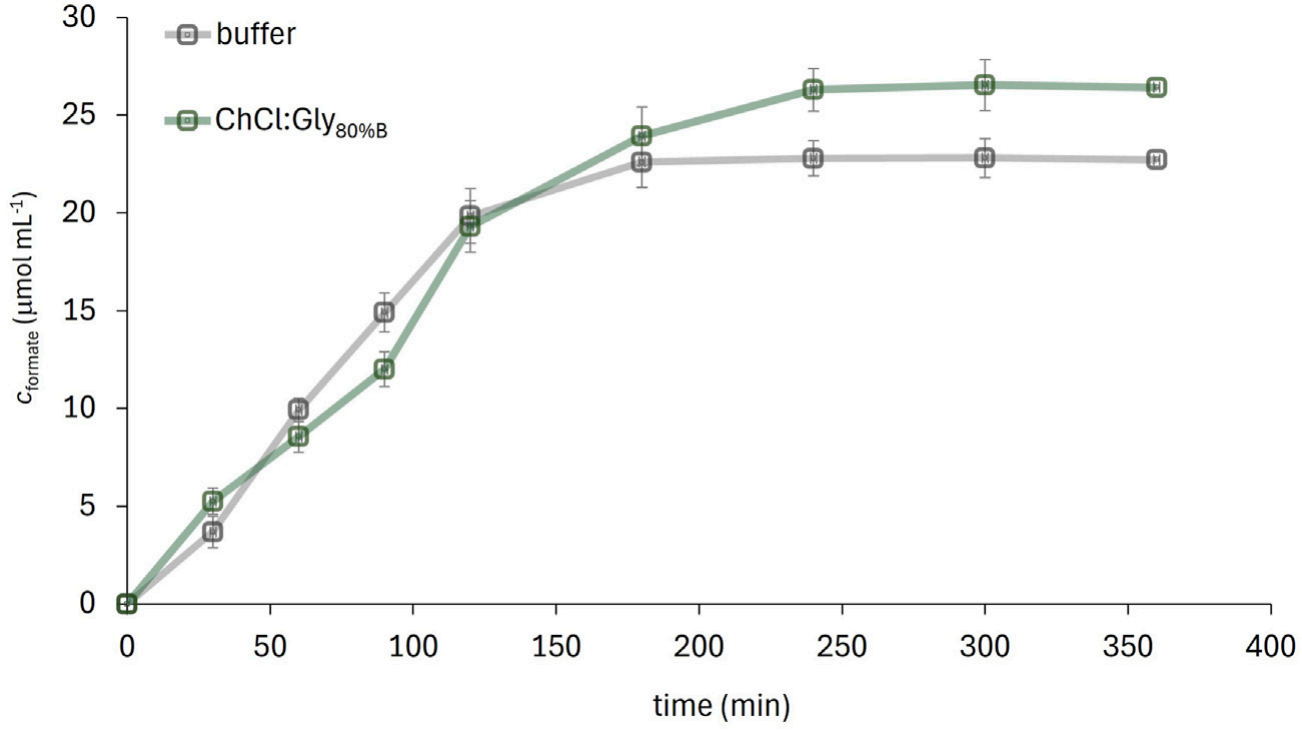
FDH-catalysed CO_2_ conversion to formate in ChCl:Gly_80%B_ (20%, w/w) solution of ChCl:Gly in potassium phosphate buffer) and the reference 50 mM potassium phosphate buffer (pH 7.5) (*c*_FDH_ = 28.8 mg mL^-1^, *c*_NADH_ = 16.4 mg mL^-1^, T = 25°C).

Since ChCl:Gly dissolved with 80% (w/w) buffer (ChCl:Gly_80%B_) was found as the most promising solvent system for the FDH-catalyzed conversion of CO_2_ to formate, the reaction was performed in a medium pre-saturated with CO_2_ and product formation was monitored over time. The amount of formate produced by the enzymatic CO_2_ reduction in the selected solvent system and in the reference buffer is shown in Figure 7. The reaction performed in the DES-supplemented medium yielded 26.5 μmol mL^−1^ of formate with a volumetric productivity of 6.6 μmol mL^−1^·h^−1^, while the reaction in the buffer yielded 22.7 μmol mL^−1^ with a volumetric productivity of 5.6 μmol mL^−1^·h^−1^. These differences can be attributed to the slightly lower solubility of CO_2_ in the buffer and the pronounced degradation of NADH in the buffer due to acidification by dissolved CO_2_ (Wu et al., 1986).

NADH stability was monitored in a separate experiment without enzyme addition: after 4 h (corresponding to the reaction time), the NADH concentration in the buffer decreased to 78% of its initial value, while in the DES medium the NADH concentration remained above 98% (data not shown). The shape of the concentration vs. time curve reflects the interplay of several factors, including enzyme activity, CO_2_ solubility and availability, and NADH degradation dynamics. The variations in formate concentration between the two solvents observed during the first 120 min may be attributed, on one hand, to the higher enzyme activity in the buffer compared to ChCl:Gly_80%B_, and, on the other hand, to the enhanced CO_2_ solubility and NADH stability provided by the DES. It is presumed that this dynamic interplay results in the reaction rate being sometimes higher in the buffer and at other times in the DES.

In addition to improving volumetric productivity, the true potential of using ChCl:Gly_80%B_ lies in its ability to stabilize FDH and thus extend the enzyme’s half-life by up to 15-fold compared to the buffer. Although this DES property is not fully utilized in a reaction lasting only 4 h, it is of great advantage in a continuous process (e.g., using an enzymatic membrane reactor). Under steady-state conditions, the volumetric productivity of the DES-assisted process could be significantly increased, as the productivity inversely correlates with the enzyme’s deactivation rate constant. Therefore, the productivity of the selected process under study was estimated for continuous operation mode comprising the enzyme inactivation rate. Considering the following assumptions: (*i*) that the process runs under steady-state conditions, (*ii*) that the enzyme is continuously deactivated over time according to first-order kinetics, and (*iii*) that the substrate concentration remains relatively constant, the reaction performed in buffer over 10-day period would yield 185.7 μmol mL^−1^ formate, while the reaction carried out in ChCl:Gly_80%B_ over the same period would yield 639.0 μmol mL^−1^ formate, which is approximately a 3.5-fold improvement. The calculation of overall productivity included estimation of the Michaelis–Menten kinetic parameters by fitting the NADH concentration profiles to the differential equation describing the change in substrate concentration over time. The results showed that both the maximum reaction rate (*v*_max_) and the NADH saturation constant (*K*_s_) were higher for the solvent system with DES. In the buffer, the constant values were *v*_max_ = 0.091 μmol mL^−1^ min^−1^ and *K*_s_ = 6.746 μmol mL^−1^, while for the systems with DES, the constants were *v*_max_ = 0.133 μmol mL^−1^ min^−1^ and *K*_s_ = 12.133 μmol mL^−1^.

Finally, downstream processing was not part of this study. Nevertheless, based on the available literature, liquid–liquid extraction with the green solvent 2-methyltetrahydrofuran (Slater et al., 2016; Laitinen et al., 2021) appears to be a promising option for the separation of dilute aqueous formate solutions in the context of developing sustainable formic acid production.

## 4 Conclusion

DESs offer considerable potential for improving biocatalytic processes. Finding the ideal solvent requires a balanced approach that considers all relevant factors, from the behavior of all reaction participants in the medium to downstream processing and the overall economic and environmental sustainability of the process. This paper presents a complementary strategy that integrates experimental screening with computational tools. By using performance targets defined by solvent properties and process constraints, this approach aims to facilitate the rational design of DESs tailored to specific biocatalytic systems. The effectiveness of the proposed approach is demonstrated using a case study of the NADH-dependent formate dehydrogenase-catalyzed reduction of CO_2_ to formate.

By systematically analyzing the performance of 20 DES-based solvents in terms of enzyme stability, activity, co-enzyme stability, and product solubility, we successfully navigated the DES screening process. It has been shown that certain DESs are highly efficient in stabilizing FDH and the coenzyme NADH, while none of the DES candidates were able to improve enzyme activity or the solubility of CO_2_. Additionally, we have demonstrated that the evaluation of different targets related to the tested reaction often leads to contradictory results regarding the optimal DES. By analyzing the data using Spearman correlation coefficients and evaluating trade-offs between the performance of DES-based solvents with respect to target properties, we identified a candidate, ChCl:Gly_80%B_, that exhibited a balanced distribution across all target property values. Moreover, this DES displayed an almost 15-fold higher FDH half-life value compared to the buffer and was more effective in stabilizing the coenzyme after addition of CO_2_. In the FDH-catalyzed reduction of CO_2_, ChCl:Gly_80%B_ outperformed the conventionally used buffered media in terms of volumetric productivity in a batch process. This solvent candidate proved to be suitable for use in continuous process due to the exceptional stability of FDH in this solvent. Furthermore, we developed a simple QSAR model to summarize the relationship between the targeted properties and the DES characteristics. These models demonstrate the feasibility of mathematical models for predicting the specific performances of DESs based on easily measurable physicochemical properties and molecular descriptors of the solvent.

However, to fully realize the benefits of DESs in biocatalysis, further research and development of computational tools and sustainable methods for product recovery is crucial. Finally, our combined experimental and computational approach increases reliability, optimizes resources, and accelerates DES-based solvent development for enzyme stabilization, as well as for other industrial applications of these green solvents. This approach improves the scalability and cost-effectiveness of DESs and represents a significant advance in DES-based industrial processes.

## 5 Materials and methods

### 5.1 Materials

For the enzymatic reactions performed in this study, the NADH-dependent formate dehydrogenase (FDH) from *Pseudomonas sp. 101* was used (see Supplementary Information) (Tishkov et al., 1993). Carbon dioxide (CO₂) with a purity of 99.5% was acquired from Messer Croatia Plin (Zaprešić, Croatia), while all other chemicals were purchased from Sigma-Aldrich (St. Louis, Missouri, United States). All materials had a purity of at least 99% and were used as received without further purification.

### 5.2 DES preparation and physicochemical characterization

For the preparation of DESs based on betaine (B) and choline chloride (ChCl), the hydrogen bond acceptor (HBA) and the hydrogen bond donor (HBD) were mixed in a molar ratio of 1:2 with water or 50 mM potassium phosphate buffer (pH 7.5) in defined proportions (Table 1). Prior to use, ChCl was dried in a vacuum concentrator at 60°C for 24 h. The mixtures were stirred and heated to 60°C until a colourless and homogeneous liquid was formed. All prepared DESs were stored in sealed bottles until further use. The pH values of the prepared DESs were measured using a pH glass electrode (Mettler Toledo, Greifensee, Switzerland). The properties of the prepared DESs (pH, density and viscosity) were determined at 25°C. Density was determined using the pycnometric method and the viscosity using a rotary viscometer (Anton Paar ViscoQC 300, Ashland, Virginia, United States). All measurements were performed in triplicates. The σ-descriptors of the DESs were calculated according to Panić et al. (2022) (Supplementary Table S1).

### 5.3 FDH activity and stability assays

To determine FDH activity, the FDH enzyme, NAD^+^ coenzyme, and formate substrate were added sequentially to various solvent systems to achieve final concentrations of 1.5 mg mL^−1^, 0.1 mg mL^−1^, and 10 mg mL^−1^, respectively. The total working volume of the assay was 250 µL. The NADH formation rate was measured immediately in a 96-well plate for 5 min at 340 nm using a UV-Vis spectrophotometer (SpectraMax^®^ ABS Plus, Molecular Devices, San Jose, CA, United States).

To evaluate FDH stability, stock solutions of the enzyme (12.8 mg mL^−1^) were prepared in the tested DES solutions and the reference buffer. These solutions were stored in sealed vials at 30°C in the dark. Aliquots were taken at regular intervals over a 14-day period and analyzed for FDH activity using the method described above.

The first-order deactivation rate constant (*k*_*FDH*_, day^-1^) was evaluated from the first-order kinetic model for the decrease of residual enzymatic activity over time (*A*_*Res*_ *(t)*) (Equation 1):

$$k_{FDH}=\frac{1}{t}\ln\left(\frac{A_{Res,0}}{A_{Res\;\left(t\right)}}\right)$$ (1)

where *A*_*Res*_ is residual enzyme activity (either at time zero, *A*_*R,*0_, or at time *t, A*_*R*_ *(t)*). The kinetic parameters were estimated by fitting the experimental data to the nonlinear equation using the Levenberg–Marquardt algorithm implemented in WR Mathematica 10.0 (Wolfram Research, Champaign, United States).

The half-life of the enzyme (*t*_*1/2, FDH*_, day) was calculated using the previously determined *k*_*FDH*_ (Equation 1), according to Equation 2:

$$t_{1/2,FDH}=\frac{\ln2}{k_{FDH}}$$ (2)

### 5.4 NADH stability assay

Measurements of the stability of the coenzyme NADH in various solvent systems (0.03 mg mL^−1^) were monitored for up to 14 days. The samples were stored in the dark at 25°C and the absorption spectra in the range from 230 to 400 nm were recorded regularly using a UV-Vis spectrophotometer (SpectraMax^®^ ABS Plus, Molecular Devices, San Jose, CA, United States). Each measurement was carried out in triplicate. The absorption spectrum of NADH shows a characteristic peak with an absorption maximum at 260 nm, which is due to the adenosine monophosphate moiety, and another peak at 340 nm, which is due to the neutral nicotinamide moiety. The decrease in absorbance at 340 nm followed first-order kinetics (Equation 3):

$$k_{NADH}=\frac{1}{t}\;\ln\left(\frac{A_{0}}{A_{t}}\right)$$ (3)

where *k*_*NADH*_ is the first-order degradation rate constant (day^-1^), *A* is the absorbance at 340 nm, either at time zero, *A*_0_, or at time *t*, *A*_t_ (Wu et al., 1986).

The NADH half-life (*t*_*1/2,*__*NADH*_, day) was calculated using the previously determined *k*_*NADH*_ (Equation 3), according to Equation 4:

$$t_{1/2,NADH}=\frac{\ln2}{k_{NADH}}$$ (4)

Moreover, the stability of NADH (25 mg mL^−1^), added in tested solvent systems after their saturation with CO_2_, was assessed to evaluate the impact of dissolved CO_2_ on the coenzyme. Monitoring was accomplished by measuring the absorbance decrease at 340 nm using a UV-Vis spectrophotometer for 90 min. The absorbance decrease at 340 nm followed the first-order kinetics, and the first-order rate degradation constants (*k*_NADH*,_ day^-1^) were calculated in the same manner as described above. For the FDH-catalyzed reduction of CO_2_ in the reference buffer and ChCl:Gly_80%B_, NADH stability was monitored throughout the entire course of the reaction (6 h).

### 5.5 CO_2_ solubility measurements

The CO_2_ solubility measurement, based on the setup described by Obert and Dave (Obert and Dave, 1999) was conducted in a glass tube where CO_2_ was bubbled through the tested solvent systems with DESs and the reference buffer (V = 5 mL) for 90 min (time sufficient to reach a saturation point) at a flow rate of 100 mL min^−1^. The gas was introduced using a small nozzle with an approximate diameter of 1 mm. To prevent CO_2_ loss, the glass tube was sealed with parafilm. The concentration of CO_2_ was continuously monitored using a Mettler Toledo (Greifensee, Switzerland) CO_2_ sensor InPro 5000i/120.

### 5.6 FDH-catalysed reduction of CO_2_

Prior to the reaction initiation, both 50 mM potassium phosphate buffer (pH 7.5) and ChCl:Gly_80%B_ were saturated with CO₂ by bubbling both solvents for 90 min, resulting in CO₂ dissolved concentrations of 1,029 mg mL^−1^ and 1,057 mg mL^−1^, respectively. The enzymatic reaction was initiated by adding NADH and FDH to the CO_2_-saturated buffer or ChCl:Gly_80%B_ to reach final concentrations of 16.4 mg mL^−1^ and 28.8 mg mL^−1^, respectively. The reactions occurred at a room temperature using a magnetic stirrer in small tubes with a working volume of 250 µL. Aliquots were sampled at specific intervals to monitor NADH consumption, which was measured spectrophotometrically at 340 nm, indicating enzyme activity in reducing CO₂. To validate the results and assess the stability of NADH under experimental conditions, control reactions were conducted without the FDH enzyme. These control mixtures allowed us to monitor NADH stability in both the buffer and ChCl:Gly_80%B_ throughout the reaction period, as described in Section 5.4. To verify that the consumed NADH was utilized for formate synthesis, the concentration of formate was additionally determined using the method described by Lang and Lang (Singh et al., 2018). Briefly, samples (25 µL) containing formate were mixed with 50 µL of solution A, 2.5 µL of solution B, and 175 µL of 100% acetic anhydride. The mixture was incubated at 50°C for 2 h with occasional mixing. Formation of red color was subsequently measured spectrophotometrically at 515 nm using the SpectraMax^®^ ABS Plus (Molecular Devices, San Jose, CA, United States). Solution A was prepared by dissolving 0.5 g of citric acid and 10 g of acetamide in 100 mL of isopropanol; solution B was prepared by dissolving 30 g of sodium acetate in 100 mL of water. For standard calibration, sodium formate dissolved in 50 mM potassium phosphate buffer (pH 7.5) was used.

### 5.7 Piecewise linear regression modelling

It was assumed that FDH activity, FDH and NADH stability can be described as a function of the DES physical properties and *σ*-profile of the mixture, expressed by a set of *S* ^*i*^_mix_ descriptors:

$$A_{R}\text{ or }t_{1/2,FDH}\text{ or }t_{1/2,NADH}=f\left({\rho ,\eta ,pH,S}_{\text{mix}}^{1},S_{\text{mix}}^{2},S_{\text{mix}}^{3},S_{\text{mix}}^{4},S_{\text{mix}}^{5},S_{\text{mix}}^{6},S_{\text{mix}}^{7},S_{\text{mix}}^{8},S_{\text{mix}}^{9},S_{\text{mix}}^{10}\right)$$

Piecewise linear regression (PLR) models have been used to describe the relationship between input and output variables. Input variables were selected based on the Spearman correlation matrix (Equation 5).

$$\begin{array}{l} A_{R}\text{ or }t_{1/2,FDH}\text{ or }t_{1/2,NADH}\\ =(b_{01}+b_{11}\cdot \rho +b_{21}\cdot \eta +b_{31}\cdot pH\\ +b_{41}\cdot S_{\text{mix}}^{1}+b_{51}\cdot S_{\text{mix}}^{2}+b_{61}\cdot S_{\text{mix}}^{3}+b_{71}\cdot S_{\text{mix}}^{4}\\ +b_{81}\cdot S_{\text{mix}}^{5}+b_{91}\cdot S_{\text{mix}}^{6}+b_{101}\cdot S_{\text{mix}}^{7}+b_{111}\\ \cdot S_{\text{mix}}^{8}+b_{121}\cdot S_{\text{mix}}^{9}+b_{131}\cdot S_{\text{mix}}^{10})\\ \cdot \left(A_{R}\text{ or }t_{1/2,FDH}\text{ or }t_{1/2,NADH}\leq b_{n}\right)\\ +(b_{02}+b_{12}\cdot \rho +b_{22}\cdot \eta +b_{32}\cdot pH\\ +b_{42}\cdot S_{\text{mix}}^{1}+b_{52}\cdot S_{\text{mix}}^{2}+b_{62}\cdot S_{\text{mix}}^{3}+b_{72}\\ \cdot S_{\text{mix}}^{4}+b_{82}\cdot S_{\text{mix}}^{5}+b_{92}\cdot S_{\text{mix}}^{6}+b_{102}\\ \cdot S_{\text{mix}}^{7}+b_{112}\cdot S_{\text{mix}}^{8}+b_{122}\cdot S_{\text{mix}}^{9}+b_{132}\cdot S_{\text{mix}}^{10})\\ \cdot \left(A_{R}\text{ or }t_{1/2,FDH}\text{ or }t_{1/2,NADH}>b_{n}\right) \end{array}$$ (5)

The PLR technique is based on estimating the parameters of two linear regression equations: one for dependent variable values (*Y*) less than or equal to the breakpoint (*b*_n_) and the other for dependent variable values (*Y*) higher than the breakpoint. The PLR parameters were estimated using the Levenberg-Marquardt algorithm implemented in the software Statistica 14.0 (Tibco Software Inc., Palo Alto, United States). The data set (63 data points for each output variable) was randomly split 70:30 into a calibration and a prediction data set. The applicability of the developed calibration models was estimated using the coefficient of determination for calibration (*R*_cal_^2^), the adjusted coefficient of determination for calibration (*R*_*cal*_^*2*^_*adj*_), and cosmo (*RMSEC*). Predictive performance of the models was estimated using the coefficient of determination for prediction (*R*_pred_^2^), the adjusted coefficient of determination for calibration (*R*_pred_^2^_adj_), the root mean square error of prediction (*RMSEP*), the ratio of prediction to deviation (*RPD*) and the ratio of the error range (*RER*) (Fearn, 2002).

### 5.8 Estimation of the productivity of the biocatalytic process

The productivity of the FDH-catalysed CO_2_ conversion to formate was estimated based on the enzyme reaction kinetics including the enzyme inactivation rate (Bisswanger, 2017). Michaelis–Menten kinetic parameters were estimated by fitting the NADH concentration profiles to the differential equation (Equation 6) using WR Mathematica 10.0 (Wolfram Research, Champaign, United States). The change in substrate concentration over time without enzyme inactivation reads:

$$\frac{dc_{NADH}}{dt}=-\frac{v_{\max}\cdot c_{NADH}}{K_{S}+c_{NADH}}$$ (6)

Considering enzyme inactivation, the following expression for the reaction rate *v(t)* (Equation 7) is obtained:

$$v\left(t\right)=\frac{v_{\max}\cdot c_{NADH}}{K_{S}+c_{NADH}}\cdot e^{-k_{FDH}\cdot t}$$ (7)

The productivity of the FDH-catalyzed CO_2_ conversion to formate can be calculated by integrating (Equation 7) from the beginning of the reaction (*t =* 0) to its end (*t = t*_*f*_) to obtain (Equation 8):

$$productivity=\int_{0}^{t_{f}}\frac{v_{\max}\cdot c_{NADH}}{K_{S}+c_{NADH}}\cdot e^{-k_{FDH}\cdot t}dt=\frac{v_{\max}\cdot c_{NADH}}{K_{S}+c_{NADH}}\cdot \left(\frac{1-e^{-k_{FDH}\cdot t_{f}}}{k_{FDH}}\right)$$ (8)

## Data Availability

The original contributions presented in the study are included in the article/Supplementary Material, further inquiries can be directed to the corresponding author.

## Glossary

**DES**: Deep eutectic solvent
**FDH**: Formate dehydrogenase
**NADH**: Nicotinamide adenine dinucleotide
**HBA**: Hydrogen bond acceptor
**HBD**: Hydrogen bond donor
**ChCl**: Choline chloride
**B**: Betaine
**Gly**: Glycerol
_**%W**_: % (w/w) of water
_**%B**_: % (w/w) of buffer
***η***: Dynamic viscosity
***ρ***: Density
***S^i^_mix_***: *σ*-profile of the mixture
**COSMO-RS**: Conductor-like Screening Model for Real Solvents
**QSPR**: Quantitative structure property relationships
***c_s_***: concentration of dissolved CO_2_
***A_R_***: relative enzyme activity
***A_Res_***: residual enzyme activity
***t_1/2, FDH_***: FDH half-life
***t_1/2, NADH_***: NADH half-life
***k_FDH_***: first-order degradation rate constant of FDH
***k_NADH_***: first-order degradation rate constant of NADH
***k_NADH*_***: first-order degradation rate constant of NADH in CO_2_ saturated solutions
***v*_max_**: maximum reaction rate
***K*_s_**: NADH saturation constant
***v(t)***: reaction rate
**PLR**: piecewise linear regression
**R*_pred_^2^***: coefficient of determination for prediction
**R*_pred_^2^*_adj_**: adjusted coefficient of determination for prediction
***RMSEP***: root mean square error of prediction
***RPD***: ratio of prediction to deviation
***RER***: ratio of the error range
***R_cal_^2^***: coefficient of determination for calibration
***R_cal_^2^*_adj_**: adjusted coefficient of determination for calibration
***RMSEC***: root mean square error for calibration
