# Supplementary material for: Harnessing the potential of deep eutectic solvents in biocatalysis: design strategies using CO2 to formate reduction as a case study
Source: Front Chem. 2024 Oct 25;12:1467810. doi: 10.3389/fchem.2024.1467810 (PMC11543487; doi:10.3389/fchem.2024.1467810)
Supplement: Supplementary file 1 [file DataSheet1.DOCX]

Supplementary Material

# Formate Dehydrogenase 101 (FDH 101) from *Pseudomonas sp. 101* production

Formate Dehydrogenase 101 (FDH 101) from *Pseudomonas sp. 101* has previously been described in: V.I. Tishkov, A.G. Galkin, G.N. Marchenko, O.A. Egorova, D.V. Sheluho, L.B. Kulakova, L.A. Dementieva, A.M. Egorov, Biochem. Biophys. Res. Commun., Volume 192, Issue 2, 1993, Pages 976-981.

The gene was ordered and obtained from Geneart in a pET28a(+) vector.

The FDH was overexpressed in *E. coli* BL21(DE3) in LB-medium (500 mL) with kanamycin (50 µg/mL final concentration) and inoculated with ONC (5 mL). Incubation was performed at 37 °C with 120 rpm in a baffled flask until an OD of 0.5 – 0,7 was reached and the expression was induced by the addition of IPTG (1 mM final concentration). After incubation overnight at 30 °C with 120 rpm, cells were harvested by centrifugation (5000 rpm, 4 °C, 20 min) and washed with buffer (TRIS-HCl, 50 mM, pH 7.5, 20 mL) followed by centrifugation (5000 rpm, 4 °C, 20 min). The cell pellets were resuspended in buffer (TRIS-HCl, 50 mM, pH 7.5, 20 mL). Cell lysis was performed via sonication (2.5 min, 30% amplitude, 2.0 sec on, 4.0 sec off). The solution was centrifuged (20 min,18000 rpm, 4 °C). The cell free extract was lyophilized and stored at 4 °C.

DNA sequence of the formate dehydrogenase *(pEG 519)*

Gene sequence, codon-optimised for expression in *E. coli*

catATGGCAAAAGTTCTGTGTGTTCTGTATGATGATCCGGTTGATGGTTATCCGAAAACCTATGCACGTGATGATCTGCCGAAAATCGATCATTATCCTGGTGGTCAGACCCTGCCGACACCGAAAGCAATTGATTTTACACCGGGTCAGCTGCTGGGTAGCGTTAGCGGTGAACTGGGTCTGCGTAAATATCTGGAAAGCAATGGTCATACCCTGGTTGTTACCAGCGATAAAGATGGTCCGGATAGCGTTTTTGAACGTGAACTGGTTGATGCCGATGTTGTTATTAGCCAGCCGTTTTGGCCTGCATATCTGACACCGGAACGTATTGCAAAAGCCAAAAATCTGAAACTGGCACTGACCGCAGGTATTGGTAGCGATCATGTTGATCTGCAGAGCGCAATTGATCGTAATGTTACCGTTGCAGAAGTGACCTATTGTAATAGCATTAGCGTTGCCGAACATGTGGTTATGATGATTCTGAGCCTGGTTCGTAATTATCTGCCGAGCCATGAATGGGCACGTAAAGGTGGTTGGAATATTGCAGATTGTGTTAGCCATGCCTATGATCTGGAAGCCATGCATGTTGGCACCGTTGCCGCAGGTCGTATTGGTCTGGCAGTTCTGCGTCGTCTGGCTCCGTTTGATGTTCATCTGCATTATACCGATCGTCATCGTCTGCCGGAAAGCGTTGAAAAAGAACTGAATCTGACCTGGCATGCAACCCGTGAAGATATGTATCCGGTTTGTGATGTTGTGACCCTGAATTGTCCGCTGCATCCGGAAACCGAACACATGATTAATGATGAAACCCTGAAACTGTTTAAACGCGGTGCCTATATTGTTAATACCGCACGTGGTAAACTGTGTGATCGTGATGCAGTTGCACGTGCCCTGGAAAGCGGTCGCCTGGCAGGTTATGCCGGTGATGTTTGGTTTCCGCAGCCTGCACCGAAAGATCATCCGTGGCGTACCATGCCGTATAATGGTATGACACCGCATATTAGCGGTACAACCCTGACCGCACAGGCACGTTATGCAGCAGGCACCCGTGAAATTCTGGAATGTTTTTTTGAAGGTCGTCCGATTCGTGATGAATATCTGATTGTTCAAGGTGGTGCACTGGCAGGTACAGGTGCACATAGCTATAGCAAAGGTAATGCAACCGGTGGTAGCGAAGAAGCAGCAAAATTCAAAAAAGCCGTGAAGCTTTAActcgag

Amino acid sequence of the Formate dehydrogenase *(pEG 519)*

# MAKVLCVLYDDPVDGYPKTYARDDLPKIDHYPGGQTLPTPKAIDFTPGQLLGSVSGELGLRKYLESNGHTLVVTSDKDGPDSVFERELVDADVVISQPFWPAYLTPERIAKAKNLKLALTAGIGSDHVDLQSAIDRNVTVAEVTYCNSISVAEHVVMMILSLVRNYLPSHEWARKGGWNIADCVSHAYDLEAMHVGTVAAGRIGLAVLRRLAPFDVHLHYTDRHRLPESVEKELNLTWHATREDMYPVCDVVTLNCPLHPETEHMINDETLKLFKRGAYIVNTARGKLCDRDAVARALESGRLAGYAGDVWFPQPAPKDHPWRTMPYNGMTPHISGTTLTAQARYAAGTREILECFFEGRPIRDEYLIVQGGALAGTGAHSYSKGNATGGSEEAAKFKKAVKL

# Supplementary Table S1. List of *σ*-profiles, DESs and water molecular descriptors

|  | **DES σ-profiles** | | | | | | | | | |
| --- | --- | --- | --- | --- | --- | --- | --- | --- | --- | --- |
|  | **S^1^** | **S^2^** | **S^3^** | **S^4^** | **S^5^** | **S^6^** | **S^7^** | **S^8^** | **S^9^** | **S^10^** |
| water | 0.000 | 6.350 | 10.027 | 3.520 | 2.164 | 2.873 | 4.055 | 5.229 | 8.277 | 0.578 |
| B:Gly_10%W_ | 0.005 | 5.652 | 10.974 | 21.584 | 22.626 | 8.235 | 6.377 | 10.666 | 12.207 | 1.914 |
| B:Gly_30%W_ | 0.003 | 6.016 | 10.479 | 12.150 | 11.940 | 5.434 | 5.164 | 7.826 | 10.154 | 1.216 |
| B:Gly_50%W_ | 0.001 | 6.178 | 10.260 | 7.968 | 7.203 | 4.193 | 4.627 | 6.568 | 9.244 | 0.907 |
| B:Gly_80%W_ | 0.000 | 6.301 | 10.093 | 4.775 | 3.585 | 3.245 | 4.216 | 5.606 | 8.550 | 0.670 |
| B:Gly_90%W_ | 0.000 | 6.328 | 10.057 | 4.091 | 2.811 | 3.042 | 4.128 | 5.401 | 8.401 | 0.620 |
| B:Gly_10%B_ | 0.005 | 5.652 | 10.974 | 21.584 | 22.626 | 8.235 | 6.377 | 10.666 | 12.207 | 1.914 |
| B:Gly_30%B_ | 0.003 | 6.016 | 10.479 | 12.150 | 11.940 | 5.434 | 5.164 | 7.826 | 10.154 | 1.216 |
| B:Gly_50%B_ | 0.001 | 6.178 | 10.260 | 7.968 | 7.203 | 4.193 | 4.627 | 6.568 | 9.244 | 0.907 |
| B:Gly_80%B_ | 0.000 | 6.301 | 10.093 | 4.775 | 3.585 | 3.245 | 4.216 | 5.606 | 8.550 | 0.670 |
| B:Gly_90%B_ | 0.000 | 6.328 | 10.057 | 4.091 | 2.811 | 3.042 | 4.128 | 5.401 | 8.401 | 0.620 |
| ChCl:Gly_10%W_ | 0.005 | 5.671 | 11.807 | 22.499 | 21.631 | 8.309 | 6.995 | 12.632 | 13.637 | 0.246 |
| ChCl:Gly_30%W_ | 0.002 | 6.033 | 10.858 | 12.380 | 11.252 | 5.411 | 5.428 | 8.685 | 10.779 | 0.423 |
| ChCl:Gly_50%W_ | 0.001 | 6.188 | 10.451 | 8.041 | 6.801 | 4.168 | 4.755 | 6.992 | 9.553 | 0.499 |
| ChCl:Gly_80%W_ | 0.000 | 6.305 | 10.146 | 4.786 | 3.462 | 3.235 | 4.251 | 5.722 | 8.634 | 0.555 |
| ChCl:Gly_90%W_ | 0.000 | 6.329 | 10.081 | 4.095 | 2.754 | 3.037 | 4.144 | 5.453 | 8.439 | 0.567 |
| ChCl:Gly_10%B_ | 0.005 | 5.671 | 11.807 | 22.499 | 21.631 | 8.309 | 6.995 | 12.632 | 13.637 | 0.246 |
| ChCl:Gly_30%B_ | 0.002 | 6.033 | 10.858 | 12.380 | 11.252 | 5.411 | 5.428 | 8.685 | 10.779 | 0.423 |
| ChCl:Gly_50%B_ | 0.001 | 6.188 | 10.451 | 8.041 | 6.801 | 4.168 | 4.755 | 6.992 | 9.553 | 0.499 |
| ChCl:Gly_80%B_ | 0.000 | 6.305 | 10.146 | 4.786 | 3.462 | 3.235 | 4.251 | 5.722 | 8.634 | 0.555 |
| ChCl:Gly_90%B_ | 0.000 | 6.329 | 10.081 | 4.095 | 2.754 | 3.037 | 4.144 | 5.453 | 8.439 | 0.567 |
